# Phasic and Tonic Pain Differentially Impact the Interruptive Function of Pain

**DOI:** 10.1371/journal.pone.0118363

**Published:** 2015-02-19

**Authors:** Christopher Sinke, Katharina Schmidt, Katarina Forkmann, Ulrike Bingel

**Affiliations:** 1 Department of Neurology, University Medical Center Hamburg-Eppendorf, Hamburg, Germany; 2 Department of Neurology, Essen University Hospital, Essen, Germany; Birkbeck, University of London, UNITED KINGDOM

## Abstract

The interruptive effect of painful experimental stimulation on cognitive processes is a well-known phenomenon. This study investigated the influence of pain duration on the negative effects of pain on cognition. Thirty-four healthy volunteers performed a rapid serial visual presentation task (RSVP) in which subjects had to detect (visual detection task) and count the occurrence of a target letter (working memory task) in two separate sessions while being stimulated on the left volar forearm with either short (2 sec) or long (18 sec) painful heat stimuli of equal subjective intensity. The results show that subjects performed significantly worse in the long pain session as indexed by decreased detection and counting performance. Interestingly, this effect on performance was also observed during control trials of the long pain session in which participants did not receive any painful stimulation. Moreover, subjects expected long painful stimulation to have a greater impact on their performance and individual expectation correlated with working memory performance. These findings suggest that not only the length of painful stimulation but also its expected ability to impair cognitive functioning might influence the interruptive function of pain. The exact relevance of expectation for the detrimental effects of pain on cognitive processes needs to be explored in more detail in future studies.

## Introduction

Pain naturally induces avoidance behavior to reduce current harm or prevent the individual from further injury and therefore ensures our survival [1]. To achieve this function, pain inherently attracts and demands attention and tends to interfere with ongoing cognitive processes resulting in detrimental cognitive performance. This phenomenon is referred to as the “interruptive function of pain” [2] and has been demonstrated for acute, experimental [3] and clinical pain [4] as well as chronic pain states [5,6]. Interestingly, as pain perception itself is highly variable, also its interruptive effect is not completely determined by the physical features of the stimulus. The detrimental effect of pain is substantially modulated by various bottom-up and top-down factors including stimulus intensity [7], stimulus novelty [8,9], stimulus predictability [8], the tendency to catastrophize about pain [10,11] and anxiety or pain-related threat [12–15]. Another modulating factor of the interruptive effect of pain is task difficulty [7,16]. Performance deficits are proposed to result from pain and task processing resources exceeding the limited capacity of attention [4,17]. While recent studies have demonstrated that this interruptive effect of pain is indeed specific for pain and not solely determined by the aversiveness of the stimuli [18] and started to unravel the underlying neural mechanisms [16,19,20], surprisingly little is known about how temporal characteristics of the painful stimulus modulate its effect on attention and task performance. As the interruptive function of pain is normally discussed in the context of limited cognitive resources [21,22], length of stimulation might be an important factor as longer stimulation might capture more processing resources. It is therefore possible that long tonic painful stimulation (e.g. chronic or recurrent pain) due to its higher cognitive load cannot be suppressed as easily as short phasic painful stimulation due to a lower cognitive load. In addition, phasic and tonic pain might have different biological functions. Short phasic pain might predominantly act as an arousal signal to closely pay attention to the environment. If pain persists, a reinterpretation from arousal to warning could switch the attentional focus from the environment towards the site of stimulation in order to escape the pain, resulting in a drop in performance. To date, several studies investigated the interruptive effect of long tonic painful stimulation on cognitive functions (e.g. [16,23,24]) while other studies applied short phasic pain stimuli to answer the same question (e.g. [3,18,20]). Importantly, no study included both phasic and tonic stimuli in a within-subject design to compare the interruptive effects of long and short pain stimuli in the same subjects.

The aim of the study therefore was to investigate the influence of stimulus duration on the interruptive function of pain. Based on previous findings showing disruptive effects of experimental pain on visual processing [16,18,20,25], we used a rapid serial visual presentation paradigm (RSVP) to test the influence of short (2 sec) and long (18 sec) painful thermal stimuli on task performance, as indexed by detection performance and working memory. In order to control for differential effects of cognitive load, task difficulty was individually calibrated in adjusting the presentation speed of the visually presented items. Further, short and long pain stimuli were matched for pain intensity. To explore possible influences of subjects’ expectation we assessed expectation ratings for the influence of long and short pain. We hypothesized the tonic painful stimulation to induce stronger impairments in visual task performance as well as well as memory performance compared to phasic painful stimuli.

## Methods

### Subjects

Thirty-four healthy participants (18 male), aged between 18 and 41 (age M ± SD: 24.7 ± 4.5 years) took part in the study. All participants were right-handed and had normal or corrected-to-normal vision. Participants had no known history of neurological or psychiatric diseases, including recurrent or chronic pain and did not take any pain medication 24 h prior to their study participation. Furthermore, all participants had normal heat pain thresholds (M ± SD 44.5°C ± 1.8°C) at the site of stimulation [26] as measured using the method of limits (see paragraph *Preparation Procedure*—Heat stimuli). The study was conducted in accordance with the Declaration of Helsinki and had been approved by the ethic commission of the medical association Hamburg. All participants gave written informed consent to participate, were free to withdraw from the study at any time and received a small monetary recompense.

### Experimental Design

**Fig 1:**
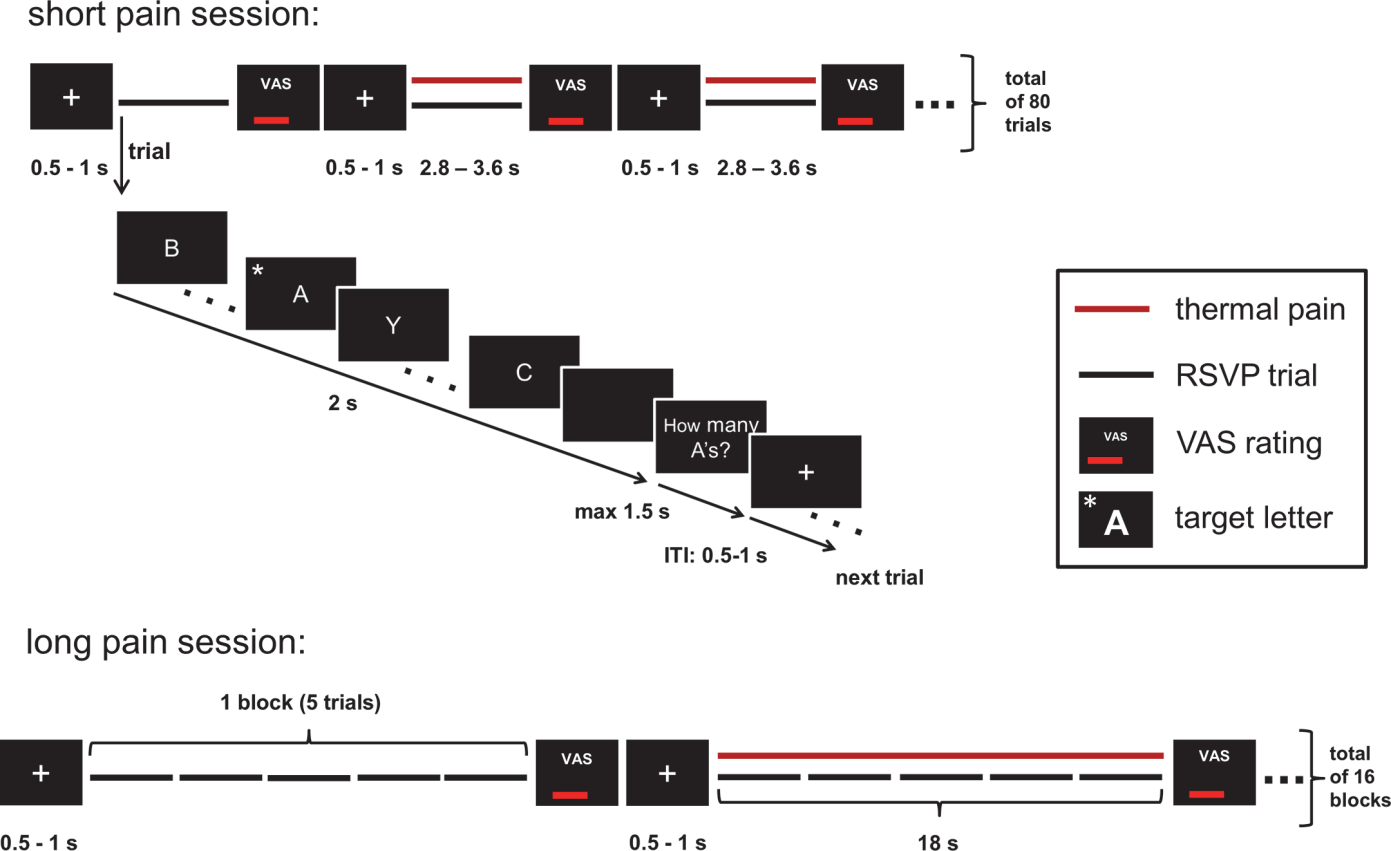
Experimental procedure. The experiment was divided into two sessions. In one session short painful stimulation was applied over 2 sec while in the other session long painful stimulation was applied over 18sec while subjects had to perform a rapid serial visual presentation task (RSVP). In this task, subjects detected the target letter ‘A’ within a stream of letters presented in an individually calibrated presentation speed. Detection was indicated by pressing the left mouse button as fast as possible (detection task). In addition subjects were asked to memorize and indicate the appearance of the target letter ‘A’ (1, 2 or 3) presented within one trial using the numbers on the keyboard (counting task). Within the long pain session painful stimulation comprised five consecutive trials, while short painful stimulation comprised only one trial. Pain intensity ratings were obtained following every painful and non-painful control trial.

All participants were asked to perform a rapid serial visual presentation task (RSVP) during which either short or long heat pain stimuli were applied to the left volar forearm. In this task a stream of letters was presented visually at a fast presentation speed that had been individually calibrated in a previous calibration phase (letter duration range: 83–117ms). Subjects were instructed to identify the target letter ‘A’ in the stream of letters and to memorize the number of targets presented within one trial (for details on session and trial structure see section RSVP Task). In order to examine the influence of pain duration on task performance either short or long noxious stimuli were applied simultaneously with the stream of visual stimuli (see Fig. 1). Thus, we employed a behavioral paradigm using a 2 × 2 factorial design with the within-subject factors PAIN (pain/no-pain) and DURATION (long/short). Importantly, the factor DURATION was investigated in two separate sessions (long pain trials + non-painful control trials and short pain trials + non-painful control trials). While trials with short painful stimulation (2 sec) and non-painful control trials (2 sec) were randomly presented in one session *(short pain session)*, the long pain session comprised trials with long painful stimulation (18 sec) and non-painful control trials (18 sec). Both sessions were separated by a short break. The order of sessions was counter-balanced between subjects to control for learning effects. At the beginning of each session subjects were asked to rate how they expected the painful stimulation to influence their task performance. To ensure comparable task performance across participants at baseline (control trials), we individually adjusted the presentation speed of letters in a calibration procedure prior to the task (see *Preparation Procedure*—Task Preparation). Moreover, long and short painful stimuli were individually calibrated to yield a comparable subjective pain level (see *Preparation Procedure*—Heat stimuli).

### Preparation Procedure

**Psychological Questionnaires**. After reading the written instructions all subjects filled in a number of questionnaires assessing control variables such as general anxiety using the *State Trait Anxiety Inventory* (STAI, [27], German version [28]), as well as pain-related anxiety and pain catastrophizing assessed with the *Pain Anxiety Symptoms Scale* (PASS-D, [29], German version [30]), and the *Pain Catastrophizing Scale* (PCS, [31], German version [32]).

Afterwards, all participants underwent a number of preparatory procedures including the assessment of heat pain thresholds, the determination of stimulation temperatures for short and long painful stimulation and the calibration of letter presentation speed.

**Heat Stimuli**. Heat stimuli were applied with a Peltier-Thermode (Cheps Pathway, Medoc, Israel) that was attached to the left volar forearm (~12 cm proximally from the wrist; 27 mm diameter stimulation area) using a bandage. The baseline temperature was set to 35°C. Heating and cooling rates for the heat pain stimuli applied during the calibration phase and the RSVP task were set to maximum (70°C/s and 40°C/s, resp.).

At first, individual heat pain thresholds at the site of stimulus application were determined using the method of limits [33]. We therefore applied ramped stimuli starting at a baseline of 35°C with 1°C/s increase in temperature and an upper limit of 50°C to avoid tissue damage. Participants were asked to indicate the first painful sensation by button press. This procedure was repeated 5 times. The mean temperature was determined as the individual heat pain threshold out of the last 4 temperature levels.

Subsequently, long and short heat pain stimuli were individually calibrated using a previously developed calibration procedure ([18] to ensure that the pain intensity was comparable between conditions and participants. This procedure aims at determining the individual temperature levels corresponding to a perceived intensity of 70 on a visual analogue scale (VAS, anchors: 0, “not painful at all”; 100, “unbearably painful”) separately for both, long and short painful stimuli. In order to avoid tissue damage and habituation to heat pain, the thermode was moved by approximately 3 cm between calibration procedures. Various temperatures near the individual heat pain threshold (short pain stimuli: -1°C < pain threshold < + 3.5°C, long pain stimuli: -2°C < pain threshold < + 1.5°C, temperature difference of 0.5°C, each temperature was applied twice) were applied in a pseudo-randomized order to the subjects’ site of stimulation. Stimulation duration at the plateau was set to 18 sec for long and 2 sec for short pain stimuli. Following each stimulus, participants gave a pain intensity rating on the VAS, which was presented on a computer screen. Subjects indicated the intensity of a stimulus by moving a red bar between the two anchors of the VAS using two mouse buttons. The temperatures that corresponded to a pain intensity level of VAS 70 were calculated from the ratings provided by a linear regression analysis. This procedure was performed independently for long and short pain stimuli and yielded mean temperatures of 47.1°C ± 1.4°C (M ± SD) for short heat pain stimuli and 45.3°C ± 1.3°C (M ± SD) for long heat pain stimuli. Prior to each experimental session subjects were asked to rate three painful stimuli in order to confirm the calibration procedure. During control trials subjects were stimulated with 35°C (baseline temperature of the Peltier-Thermode). The software Presentation (Neurobehavioral Systems, Inc., Albany, CA) was used to trigger painful stimulation and to present the visual stimuli and the VAS on a Dell latitude E5430 laptop computer. Moreover, Presentation automatically recorded all behavioral data.

**Task Preparation**. The difficulty of the RSVP task was individually adjusted to reach a performance level of ~80% correct detection rate. To this end, the presentation duration of a letter was set to 140 ms and was decreased by 10 ms after every 10th trial (trial length 2 sec, for details see RSVP task) until a minimal presentation speed of 80 ms per letter was reached. In total, participants performed 80 trials within this calibration phase. The individual presentation speed closest to 80% correct detection rate was chosen to be used in the experimental phase. The average presentation speed was 103 ms ± 12 ms (M ± SD, range: 83 ms—117 ms) per letter.

At the beginning of each session, expectation values about the detrimental or beneficial effects of either long or short painful stimulation were obtained using a VAS that was presented on the computer screen (- 5 to + 5; anchors labeled as “severely impaired” and “highly improved”, middle label “no effect”).

**RSVP Task**. Arabic capital letters (A—Z, without umlaut, font type “Arial”) were presented in white color on a black screen. The letters had a visual angle of ~ 4°×3° (height, width) and were presented for an individually adjusted duration (see *Preparation Procedure*—Task Preparation)) with no break between single letters.

Each of the two sessions comprised 80 trials (40 painful, 40 control) in which a stream of letters was presented over a period of 2 sec. The number of letters was dependent on the individually calibrated presentation speed with 19 ± 3 (M ± SD, range: 16–23) letters within one trial. In the short pain session brief painful stimuli (2 sec duration) were applied during a single trial, while long pain stimuli of 18 sec duration that covered five consecutive trials (1 block) were applied in the long pain session. Painful trials and the corresponding non-painful control trials were presented in random order. Furthermore, participants were not informed about the trial type presented next (painful or control). In each trial the target letter ‘A’ either appeared 1, 2 or 3 times. The position of the target letter within the presentation stream was chosen randomly with the limitation that it did not occur within the first 3 or the last 3 items of a trial and that each target letter was at least 300 ms apart from the next target to ensure participants could visually process the targets separately and perform the required motor response. The presentation duration of a single letter was individually adjusted as described above (see paragraph *Preparation Procedure*—Task Preparation)). Subjects had to perform two tasks: they had to (1) detect target letters (visual detection task) and (2) count target letters (working memory task). Subjects were instructed to press the left mouse button with the index finger of the right hand as fast as possible whenever the letter ‘A’ was presented (detection task). In addition, they were asked to memorize the number of ‘A’s presented within one trial (min 1, max 3) and indicate this number after each trial by pressing the appropriate button number on the keyboard with the left hand as fast as possible, but within 1.5 sec (counting task, see also Fig. 1A). As heat pain stimuli were applied to the left volar forearm and only right-handed volunteers participated in the study, we did not include response times of left hand button presses as an outcome variable. After completion of the counting task and at the end of the thermal stimulation, participants rated the intensity of each stimulus (painful and control) on a VAS. Following the rating and between trials a fixation cross of variable duration (500–1000 ms) was presented. See Fig. 1 for details about the trial structure and the painful stimulation.

**Analysis**. Behavioral data were automatically recorded and logged by the stimulation program Presentation and data analyses were performed using SPSS 18. Mean correct detection rate, mean reaction times (RT) to detect the target letter and mean correct counting performance were analyzed using 2×2 repeated-measurement ANOVAs including the within-subject factors PAIN (pain/no-pain) and DURATION (long/short). Mean RTs were computed for correctly identified letters separately for each experimental condition after removing extreme outliers. Reactions and detected letters with RT shorter than 100 ms or longer than 3 SDs above the individual mean RT were excluded as outliers from additional analyses (number of excluded detection trials in long session: pain 4.5 ± 2.3, control 4.3 ± 1.8; short session: pain 4.8 ± 2.3, control 3.9 ± 2.3). The individual mean detection performance was defined as the percentage of detected target letters separately in each of the two conditions. Percentage of correctly indicated number of targets was defined as the individual counting performance.

Prior to the statistical analyses all variables were tested for normal distribution using the Kolmogorow-Smirnov test and homogeneity of variance using Levene test. No violation for any dependent variable was detected.

In order to test for potential learning effects between sessions, we employed two-sample t-tests on the dependent variables and compared subjects starting with the long pain session and subjects starting with the short pain session. Mean pain ratings provided in the short and long pain session were compared using paired t-tests in order to assure that detrimental effects of pain were not driven by differences in subjective pain intensity. Furthermore, expectations about the interruptive effect of painful stimulation were compared between sessions using paired t-test. Correlations between expectation, pain-related questionnaires and task performance were calculated with Spearman’s correlation coefficient.

## Results

### Order of session

We assessed whether task performance (detection rate, reaction times of detection, counting rate) differed depending on the order of long and short pain sessions. No differences were found, neither for the detection performance (detection rate, reaction times of detection) nor the counting performance (all p > 0.32).

### VAS rating

The perceived intensity of the pain stimuli that were individually calibrated to an intensity of VAS 70 dropped during the RSVP task (F(1,33) = 61.973, p < 0.001, η^2^ = 0.653) but still yielded moderate intensity ratings (long pain stimuli: 53.25 ± 15.9 VAS; short pain stimuli 53.33 ± 18.3, M ± SD). Importantly, mean pain intensity did not differ between short and long pain sessions (t(33) = 0.02, p = 0.98).

### Detection task

**Fig 2:**
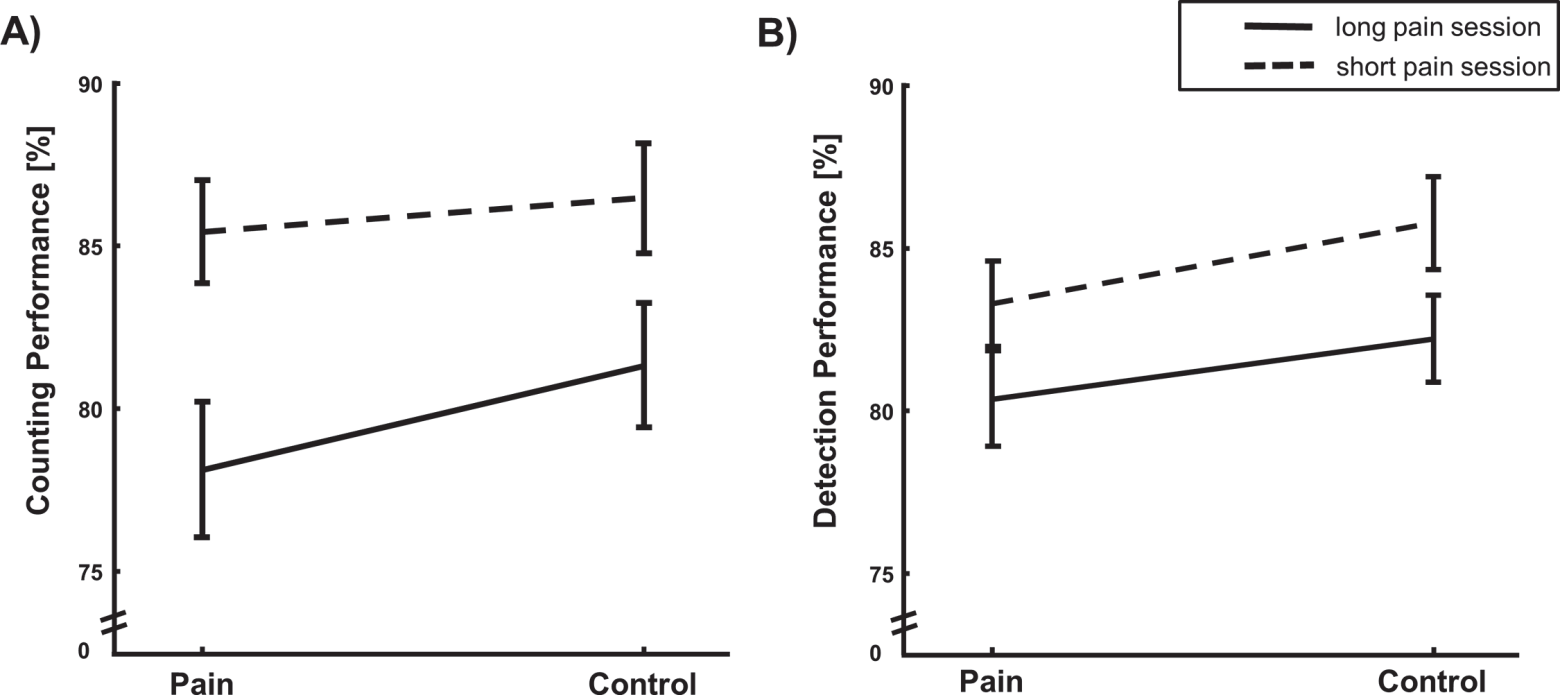
Task performance. A: Counting accuracy (M ± SEM in % correct answers) in long and short pain session. Subjects perform significantly worse under pain and significantly worse in the long pain session (main effect of PAIN and STIMULATION). B: Detection accuracy (M ± SEM in % correct detections). Performance significantly decreased with painful stimulation and in the long pain session (main effect of DURATION and STIMULATION).

The 2×2 ANOVA on detection rates yielded a significant main effect of DURATION (F(1,33) = 20.478; p < 0.001, η^2^ = 0.38) with reduced detection performance in the long pain session and a significant main effect for the factor PAIN (F(1,33) = 8.118; p = 0.007, η^2^ = 0.20; see Fig. 2B and Table 1) with reduced detection performance under pain but no interaction between both factors (F(1,33) = 0.129, p = 0.72).

**Table 1:** Behavioral data.

	Pain trials	Control trials
**Detection rate in short pain session (%)**	83.3 ± 7.7	85.8 ± 8.3
**Detection rate in long pain session (%)**	80.3 ± 8.6	82.2 ± 7.8
**Correct answers in short pain session (%)**	85.4 ± 9.2	86.5 ± 9.9
**Correct answers in long pain session (%)**	78.1 ± 12.1	81.3 ± 11.2
**RT detection in short pain session (ms)**	373 ± 26	372 ± 25
**RT detection in long pain session (ms)**	383 ± 26	380 ± 28
**VAS rating short pain session (VAS)**	53.3 ± 18.3	7.7 ± 8.4
**VAS rating long pain session (VAS)**	53.3 ± 15.9	8.3 ± 9.2

Performance in the pain and control trials for the long and short pain session and mean VAS ratings (M ± SD).

A significant main effect of DURATION (F(1,33) = 12.23; p = 0.001, η^2^ = 0.27) was revealed by the 2×2 ANOVA on reaction times with faster reaction times in the short pain session. In contrast, we found no main effect of PAIN (F(1,33) = 0.08, p = 0.38) and no interaction between PAIN and DURATION (F(1,33) = 0.23, p = 0.64).

### Counting task

The 2×2 ANOVA revealed a significant main effect of DURATION on the counting performance (F(1,33) = 33.744; p < 0.001, η^2^ = 0.51 see Fig. 2A and Table 1) with reduced counting performance in the long pain session as well as a significant influence of PAIN (F(1,33) = 4.15; p = 0.05, η^2^ = 0.11) with impaired performance under painful stimulation. No interaction of PAIN and DURATION could be detected (F(1,33) = 1.5, p = 0.23).

### Pain-related questionnaires

Results of the control variables general anxiety and pain-related anxiety as well as pain catastrophizing can be seen in Table 2. No correlations between questionnaire scores and detection as well as counting performance could be detected.

**Table 2:** Data from pain-related and anxiety-related questionnaires.

Questionnaire	Score (mean ± SD)
**STAI—A**	38.2 ± 6.7
**STAI—B**	36.7 ± 8.2
**PCS**	15.3 ± 7.7
**PASS—D1**	23.6 ± 9.5
**PASS—D2**	22.8 ± 7.8
**PASS—D3**	11.6 ± 6.7
**PASS—D4**	15.0 ± 9.9

STAI A: State anxiety; STAI B: Trait anxiety; PCS: Pain catastrophizing scale; PASS: Pain anxiety symptoms scale, D1: Fearful appraisal of pain, D2: Cognitive anxiety, D3: Physiological anxiety, D4: Escape and avoidance behavior.

### Expectation

**Fig 3:**
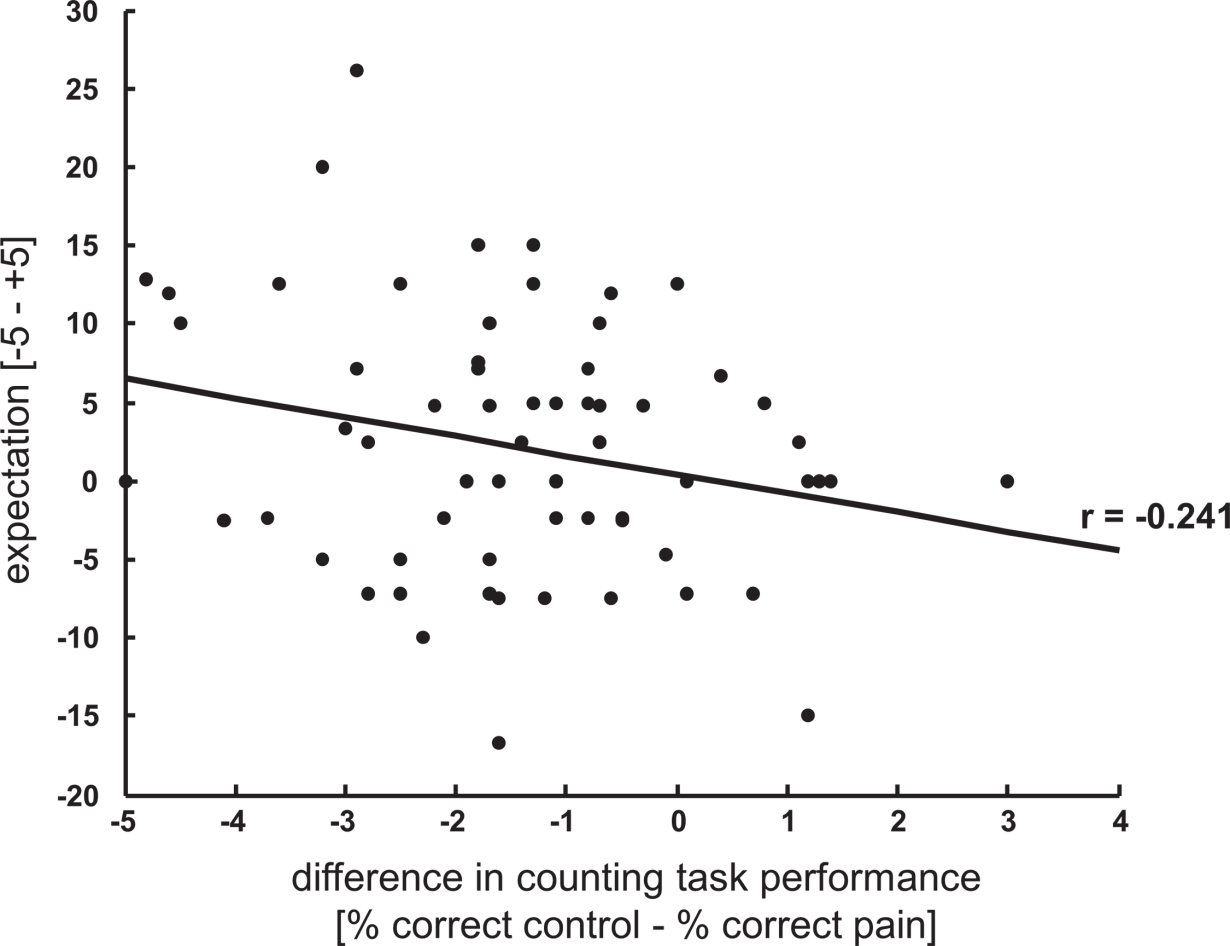
Task performance correlates with individual expectation. Correlation between the subjects’ expectation about the influence of pain on task performance and the actual performance difference in the counting task between painful and non-painful trials showing that the more subjects expect pain to disturb their performance the worse their short-term memory performance is under pain.

Subjects expected both stimuli to impair their performance (long painful stimuli: VAS -1.72 ± 1.63, short painful stimuli -1.07 ± 1.49; one-sample t-test: long painful stimuli: t(33) = 6.1, p < 0.001; short painful stimuli: t(33) = 4.2, p < 0.001). Long painful stimuli were expected to impair the performance to a higher degree (t(33) = 2.061; p = 0.047, dz = 0.35). To further explore the role of expectancy on task performance we correlated the individuals’ expectation ratings with their individual difference in task performance between control and painful trials (performance control trials—performance pain trials). Note that two values per subject (one for the long pain session and one for the short pain session) were included in this analysis. This exploratory analysis reveals a slight to moderate association between expectation and counting task performance (r = -0.241, p = 0.048, see Fig. 3) suggesting that the genuine expectation about the influence of pain on task performance actually modulated cognitive processes.

## Discussion

This study investigated the influence of pain duration on the interruptive function of pain by applying tonic or phasic painful heat stimuli during a visual detection task. The cognitive load imposed by the task led to a reduced perception of pain, as reported in previous studies (e.g. [7]. Furthermore, we show that pain disrupts detection performance in the employed RSVP task. Irrespective of its duration, pain impaired visual target detection and working memory (i.e., counting the number of targets). Visual processing and working memory have been shown to be affected by pain in previous studies [7,16,18,20,25]. Here, we aimed at extending these findings by testing the influence of stimulus duration on the interruptive function of pain. As hypothesized, the duration of painful stimulation influenced task performance. Interestingly, this influence was not specific for pain, since we could not observe an interaction between stimulation duration (long vs. short) and painful stimulation (pain vs. control) and instead found a main effect of stimulus duration. Tonic painful stimulation led to a decrease in detection performance and increased reaction times in both painful trials and non-painful control trials compared to short painful stimulation. Moreover, working memory performance in long pain sessions was also decreased compared to the performance during short pain sessions. This effect was independent of painful stimulation itself as subjects also performed differently in control trials of the long pain session compared to control trials of the short pain session, which points to a global effect influencing performance. The observed global effect is unlikely to be a result of learning processes as all subjects performed a sufficient number of exercise trials before the experiment and we did not find any differences in performance between the two different session orders.

As proposed by the Inverted-U-Theory by Yerkes & Dodson [34] performance reaches its maximum under medium levels of arousal. It is therefore tempting to speculate that although both tonic and phasic pain increased the level of arousal, phasic stimuli induced an intermediate level of arousal that did not impair performance. Conversely, tonic stimuli could have increased the arousal to a level that leads to compromised performance which might explain the observed differences in the interruptive function of pain. However, note that we did not measure arousal in the present study. Instead, we were focusing on the participants’ expectation regarding the impairing effect of long and short pain on their performance. Intriguingly, subjects expected differences in the interruptive effect of long and short pain. Specifically, the more participants expected pain to impair their performance the stronger was their individual decrease in working memory performance during pain, indicating that at least a small proportion of variance in individual behavior is explained by expectancy. It is well known that positive and negative expectations can increase or reduce the perception of pain, as best illustrated in placebo or nocebo paradigms [35,36]. In addition, anticipated pain can induce activation in pain processing areas [37]. Whether an individual’s expectation has an impact on the interruptive function of pain has not yet been investigated. One intriguing phenomenon illustrating the importance of expectation on cognitive functioning is the so called *stereotype thread* [38]. People tend to perform differently when certain stereotypes about their peer group are activated (e.g. “women are bad at math(s)” [39], “older people have a bad memory” [40]). One could speculate that the individual expectation that pain disrupts performance indeed affected the performance on the individual level in our study. One explanation for the global effects of expectation on the interruptive function of pain could be that within the stimulation sessions subjects could not predict whether the next trial would be paired with a painful heat stimulus or not. Subjects therefore did not have sufficient information to direct their expectation in a specific way. Although the order of painful and non-painful trials in the long pain session followed a certain pattern we assume that subjects were not aware of this pattern. It is therefore conceivable that this difference in expectation between long and short pain session decreased the performance during long painful and non-painful stimulation and thereby produced the observed main effect of pain duration. Interestingly, Colagiuri et al. [41] showed that expectation can influence cognitive performance. In their study subjects were told that an odor has either beneficial or detrimental effects on cognitive performance. The positively instructed group displayed faster reaction times during the experiment, in trials with odor stimulation as well as in control trials as compared to the negatively instructed group. This implies that expectation might work in a rather unspecific way. As painful stimulation was not cued, our study design was not suited to dissect between subject’s individual expectation of the interruptive function of pain and the expectation of an upcoming stimulus itself. Future studies could use cues to inform about the upcoming painful stimulation or non-painful control trials and to dissociate these effects.

Our study did not compare the effect of pain with that of other aversive stimuli (see [18]) and the experimental noxious stimuli used here only resemble chronic pain to a limited degree. Nevertheless, the phenomenon of expectation-induced differences in the interruptive function of pain might have implications for patients suffering from chronic pain, which is well known to be associated with cognitive impairment [6,18,42–44]. It is tempting to speculate that moreover, the experience of long-standing pain and cognitive dysfunctions induces strong negative expectations, which in turn enhance the impairing function of pain as pain perception as well as the expectation and anticipation of pain are inherently linked. Based on the reported results these detrimental effects of increased negative expectation could then transfer to pain-free periods as chronic pain patients might be in continuous expectation and anticipation of pain.

## Conclusion

In this study we examined the influence of pain duration on the interruptive function of pain. Overall, a multitude of nonlinear influences, such as personality traits, stimulus intensity, or stimulus novelty have been shown to modulate the interruptive function of pain [8,11,13,15,45]. In this study we could extend these findings by showing that the duration of the applied heat pain stimuli modulates the interruptive function of pain. Further research is needed to explore how the influence of duration is mediated by expectation and how our findings translate into chronic pain states.

## Supporting Information

S1 Dataset The analyzed dataset. Provided are age, gender, applied temperature, questionnaire scores, VAS ratings, detection and counting performance for each subject.
(XLSX)
